# Volatile Organic Compound Profiles of *Cystoseira corniculata* (Turner) Zanardini 1841 and *Ericaria amentacea* (C.Agardh) Molinari and Guiry 2020 (ex. *Cystoseira amentacea* (C.Agardh) Bory de Saint-Vincent, 1832)

**DOI:** 10.3390/molecules27207131

**Published:** 2022-10-21

**Authors:** Sanja Radman, Igor Jerković

**Affiliations:** Department of Organic Chemistry, Faculty of Chemistry and Technology, University of Split, Ruđera Boškovića 35, 21000 Split, Croatia

**Keywords:** headspace, volatiles, gas chromatography and mass spectrometry

## Abstract

The volatile organic compounds (VOCs) of fresh (FrCC) and air-dried (DrCC) *Cystoseria corniculata* and fresh (FrEA) and air-dried (DrEA) *Ericaria amentacea* from the Adriatic Sea were investigated by headspace solid-phase microextraction (HS-SPME) and hydrodistillation (HD) and analysed by gas chromatography and mass spectrometry (GC-MS). In HS-FrCC and HS-DrCC, aliphatic compounds were dominant, with decan-5-ol as the most abundant in HS-FrCC, but in HS-DrCC pentadecane dominated. Monoterpenes (β-cyclocitral, β-citral, and β-cyclohomocitral) and sesquiterpenes (cubenol) were abundant in HS-FrCC. Notable differences between fresh and air-dried samples were found for benzene derivatives. Fatty acids and their derivatives were the most abundant in HD-FrCC and HD-DrCC. In HS-FrEA and HS-DrEA, saturated aliphatic compounds as well as unsaturated aliphatic compounds (particularly hexan-1-ol and (*Z*)-hex-3-en-1-ol) predominantly showed decrements after drying. Pentadecane, heptadecane, pentadecanal, and hexan-1-ol were predominant in HD-FrEA, and their percentage decreased in HD-DrEA. The percentage of monoterpenes decreased after drying, but the percentages of diterpenes and especially sesquiterpenes increased. δ-Selinene was the major terpene and the most abundant in HD-DrEA. A significant increment after drying could be noticed for fatty acids and their derivatives. The great diversity of identified VOCs among these two macroalgae supports their different botanical classifications.

## 1. Introduction

*Cystoseira* C.Agardh 1820 is a genus of marine macroalgae (around 40 species) of the Sargassaceae family distributed along the Atlantic and Mediterranean coasts [1]. They produce various metabolites such as terpenes (including meroterpenoids), steroids, phlorotannins, phenolic compounds, carbohydrates, triacylglycerols/fatty acids, and pigments as well as vitamins [2,3,4]. The biological and chemical diversity of *Cystoseira* macroalgae has great potential for the discovery of novel compounds with biomedical relevance. The extracts and some isolated compounds were associated with antioxidant, anti-inflammatory, cholinesterase inhibition, anticancer, cytotoxicity, antidiabetic, antibacterial, antifungal, and antiparasitic activities [4].

There are only a few reports on the phytochemical composition of *C. corniculate*. Free amino acids and amino acids of protein fractions were extracted from *C. corniculata.* [5] The fatty acid profile of *C. corniculata* from the northeastern Mediterranean was investigated [6]. The proportion of polyunsaturated fatty acids (PUFAs)-*n*3 was 14.89%, and PUFAs-*n*6 was 13.79%. The major fatty acids were palmitic, oleic, and arachidonic acid. The protein content of *C. corniculata* from Turkey [7] was 43.72 mg g^−1^, the carbohydrate concentration was 20.74 mg g^−1^, the total phenolic content was 0.469 mg g^−1^, and the major pigments were chlorophyll *a* (0.839 mg g^−1^) and carotenes 0.154 (mg g^−1^). The total carotenoid content in this macroalga from the Adriatic Sea [8] was 1.197 mg g^−1^ (β-carotene 55.5% and fucoxanthol 45.5%). There are no data about the chemical composition of the volatile organic compounds (VOCs) of *C. corniculata*.

*Ericaria amentacea* (C.Agardh) Molinari and Guiry 2020 is currently taxonomically accepted instead of *Cystoseira amentacea* (C.Agardh) Bory de Saint-Vincent, 1832. *E. amentacea* from Sicilia, Italy, has been studied [4,9,10,11,12]. Different tetraprenyltoluquinols have been isolated with regular (strictaketal, isocystoketal, isostrictaketal, isobalearone, (*Z*,*E*)-bifurcarenone, amentaepoxide, and amentadione) or irregular (neobalearone and 2-epi-neobalearone) diterpenoid moieties. Demethoxy cystoketal chromane and cystoketal quinone (new cystoketal derivatives) were isolated from *E. amentacea* from France in addition to sterols and meroditerpenes [4], and two new compounds were found (4′-methoxy-(2*E*)-bifurcarenone and its chromene derivative). Meroditerpenes were found [13] in DMSO and 50% ethanolic extracts of this macroalga from the northwestern Mediterranean with the structures containing chromane or quinone groups, such as cystoketal quinone, demethylcystoketal chromane, cystoketal chromane, and/or cystoketal. In our previous paper, we investigated the composition of less polar fractions of *E. amentacea* from the Adriatic Sea and amides of higher aliphatic acids, carotenoids, chlorophyll derivatives (the subgroup containing 55 carbon atoms, such as pheophytin *a* and its derivatives), and higher terpenes were found [14]. To the best of our knowledge, we did not find any data about *E. crinita* VOCs.

The aim of this study was to detail investigate the volatilomes of *C. corniculata* and *E. amentacea* (formerly known as *C. amentacea*). The two species were selected from the Adriatic Sea in order to compare their volatilomes since it is known that *Cystoseira* species exhibit great biological and chemical diversity [1,2,3,4]. Therefore, we expected to find differences in the VOC profiles of these two macroalgae obtained by hydrodistillation (HD) and headspace solid-phase microextraction (HS-SPME). The use of different and complementary methods for headspace, volatile, and less volatile compounds was expected to provide new data. The present research also offers novelty regarding the influence of air drying on the volatilome. The hydrodistillate and extracts were analysed by gas chromatography and mass spectrometry (GC-MS).

## 2. Results and Discussion

To study the diversity of VOCs from *C. corniculata* and *E.* *amentacea*, both harvested from the Adriatic Sea, fresh (FrCC and FrEA) and air-dried (DrCC and DrEA) samples were analysed. Their headspace composition was analysed using solid-phase microextraction (HS-SPME). To achieve more detailed results, two fibres of different polarities were used: divinylbenzene/carboxene/polydimethylsiloxane (DVB/CAR/PDMS, f1) and polydimethylsiloxane/divinylbenzene (PDMS/DVB, f2). The volatile oil was obtained by hydrodistillation (HD).

### 2.1. Headspace Composition of C. corniculata

**Figure 1:**
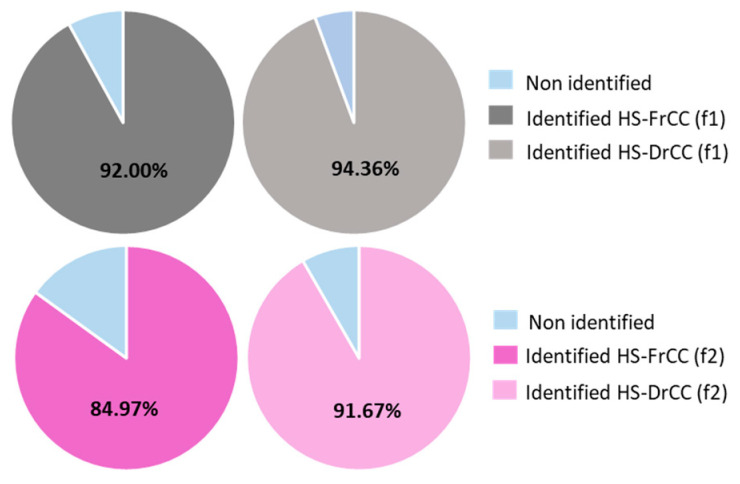
Diagram showing the portion of identified compounds in the headspace of *C. corniculata*. HS-FrCC—headspace of fresh *C. corniculata*; HS-DrCC—headspace of air-dried *C. corniculata*; f1—DVB/CAR/PDMS fibre; f2—PDMS/DVB fibre.

To this day, there is no report on the headspace chemical composition of *C. corniculta*. In FrCC headspace (HS-FrCC), 92.00% (f1) and 84.97% (f2) areas under the peaks of the chromatograms of VOCs were identified in total, while in DrCC headspace (HS-DrCC) 94.36% (f1) and 91.67% (f2) of VOCs were identified in total (Figure 1).

**Figure 2:**
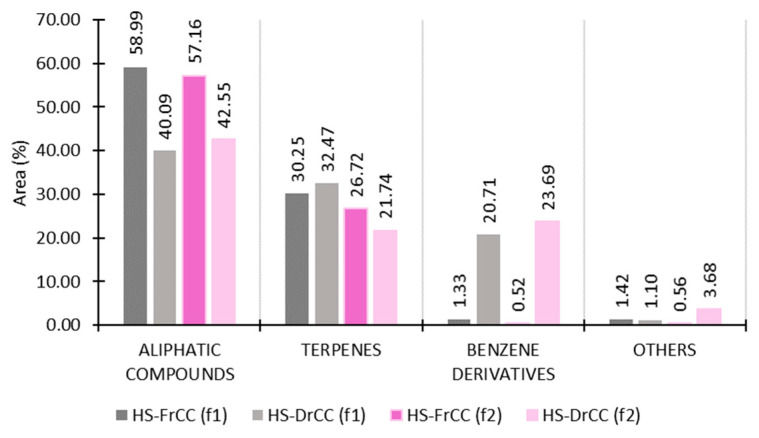
The VOCs of *C. corniculata* extracted by HS-SPME, analysed by GC-MS, and sorted by structural groups. HS-FrCC—headspace of fresh *C. corniculata*; HS-DrCC—headspace of air-dried *C. corniculata*; f1—DVB/CAR/PDMS fibre; f2—PDMS/DVB fibre.

The dominant group was aliphatic compounds in both HS-FrCC (58.99%, f1; 57.16%, f2) and HS-DrCC (40.09%, f1; 42.55%, f2) (Figure 2). Saturated aliphatic compounds prevailed, with decan-5-ol as the most abundant compound in HS-FrCC (33.56%, f1; 30.42%, f2). Its presence in marine algae is reported for the first time. More aliphatic alcohols were detected to have high abundance: hexan-1-ol (8.12%, f1; 9.78%, f2), (*Z*)-hex-3-en-1-ol (4.05%, f1; 6.24%, f2), and dec-4-en-1-ol (2.53%, f1; 2.55%, f2) (Table 1). All of the above-mentioned alcohols probably completely evaporated after air drying as a result of their high volatility. The saturated aliphatic ketone decan-5-one (4.22%, f1; 3.00%, f2) could not be detected in the sample after air drying. This ketone was not yet reported in marine algae. Literature data showed that pentadecane dominates in the brown algae [15,16], which was not the case here since in HS-FrCC it was detected in a very small percentage (<0.40% with both fibres). However, our earlier research showed that pentadecane significantly increased after drying [17,18], and similarly, in HS-DrCC, pentadecane was the most abundant compound (27.62%, f1; 21.90%, f2). This increment could be the consequence of fatty acid degradation [19]. Heptadecane was highly abundant in HS-DrCC as well (6.22%, f1; 9.19%, f2) (Table 1).

The second most abundant group in HS-FrCC was the group of terpenes (30.25%, f1; 26.72%, f2) (Figure 2), with similar percentages of monoterpenes (15.14%, f1; 13.68%, f2) and sesquiterpenes (15.12%, f1; 13.04%, f2). The aldehydes β-cyclocitral, β-citral, and β-cyclohomocitral were major components within the monoterpenes and completely evaporated after drying, probably due to their higher volatility. Among the sesquiterpenes, the most abundant was cubenol (10.37%, f1; 8.57%, f2), which decreased after drying. In HS-DrCC, there were no monoterpenes detected, only sesquiterpenes (32.47%, f1; 21.74%, f2). The difference could be noticed when comparing the analysis among the two fibres. β-Cubebene, followed by germacrene D, were the most abundant sesquiterpenes with f1, while cubenol was the most abundant when analysing with f2 (Table 1).

The largest difference between the fresh and air-dried samples was present in the group of benzene derivatives. In HS-FrCC, their content was 15.5 times lower than that extracted with f1 and even 45.2 times lower than that extracted with f2 (Figure 2). Among benzene derivatives, benzyl alcohol, phenol, and benzaldehyde were identified. A large increase in the benzaldehyde portion after drying was noted. Benzyl alcohol was the most abundant compound in the HS-DrCC extracted with f2 (21.35%) and the second most abundant in the HS-DrCC extracted with f1 (17.39%) (Table 1). The phenylpropane derivative increment could be linked to phenylalanine degradation with a side-chain shortened by two carbon atoms as a consequence of the β-oxidation (or even nonoxidation) process [20].

Among the group of other compounds, two dictyopterenes (dictyopterene C’ and D’) and dimethyl sulphide were present (Table 1).

### 2.2. Volatile Oil Composition of C. corniculata

**Figure 3:**
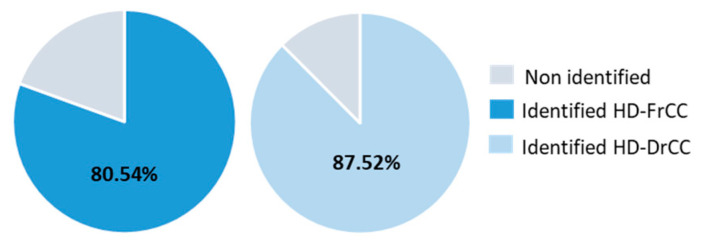
Diagram showing the portion of identified compounds in the hydrodistillate of *C. corniculata*. HD-FrCC—hydrodistillate of fresh *C. corniculata*; HD-DrCC—hydrodistillate of air-dried *C. corniculata*.

The analysis of the hydrodistillate of *C. corniculata* resulted in an 80.54% portion of identified compounds in the fresh (HD-FrCC) sample and an 87.52% portion in the air-dried (HD-DrCC) sample (Figure 3).

**Figure 4:**
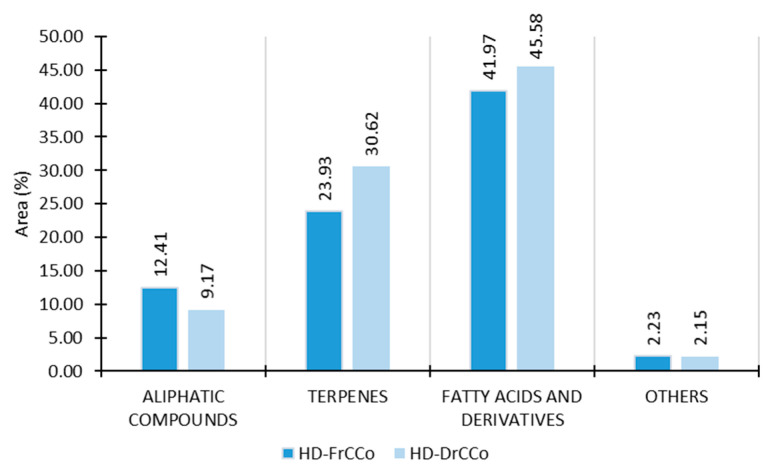
The VOCs of *C. corniculata* obtained by HD, analysed by GC-MS, and sorted by structural groups. HD-FrCC—hydrodistillate of fresh *C. corniculata*; HD-DrCC—hydrodistillate of air-dried *C. corniculata*.

Fatty acids and their derivatives were predominant in both the fresh and dry samples (Figure 4). Two fatty acid esters (FAEs), methyl eicosanoate (23.88%, HD-FrCC; 23.01%, HD-DrCC) and butyl stearate (14.12%, HD-FrCC; 13.07%, HD-DrCC) (Table 2), contributed the most. Five more FAEs were identified, with similar abundances in both the fresh and dry samples, except (*E*)-octadec-9-enoic acid methyl ester, whose content increased 11.0 times after air drying (Table 2). Fatty acid methyl esters (FAMEs) in the hydrodistillate presented 26.25% in fresh and 30.73% in dry *C. corniculata*. Periera et al. [21] analysed six species of Phaeophyta, including *Sargassum vulgare,* which belongs to the same order as *C. corniculata*: Fucales. Their research showed that the analysed pheaeophyta contained 30–45% of the total FAME [21]. In HD-FrCC, palmitic acid (hexadecanoic acid) and oleic acid ((*Z*)-octadec-9-enoic acid) were found in small portions. Polat and Ozogul [6] analysed the contents of the fatty acids in *C. corniculata*. The results showed that 31.72% of all fatty acids were monounsaturated fatty acids (MUFAs), 31.66% were saturated fatty acids (SFA), and 18.08% were polyunsaturated fatty acids (PUFAs). The most represented acids were palmitic acid, followed by oleic acid, myristoleic acid, and linoleic acid. The full profile of the fatty acids could not be isolated with hydrodistillation. Thus, it is not possible to compare those results with the results of the fatty acids isolated from the extracts.

Among the group of terpenes, diterpenes (13.74%, HD-FrCC; 16.66%, HD-DrCC) led, followed by sesquiterpenes (10.10%, HD-FrCC; 13.59%, HD-DrCC). (*E*)-Geranylgeraniol was the most abundant of all terpenes and was stable after drying. It previously occurred in *C. brachycarpa* [22], *C. balearica* [23], and *C. tamariscifolia* [24]. This diterpene alcohol could be used as a precursor for synthesizing vitamins A and E [25], and it regulates testosterone production [26]. It also induces anticancer, antitumor, and antileishmanial potentials [27]. Other terpenes that were significant in terms of abundance, such as cubenol, sesquiterpene alcohol, and diterpene alcohols (cembra-4,7,11,15-tetraen-3-ol, pachydictyol A, isopachydictyol A, and (*E*)-phytol) increased after drying (Table 2). In *C. stricta* var. *amentacea,* cubenol was the major constituent [28]. This alcohol is one of the compounds responsible for the typical “ocean smell”. Pachydictyol A and its isomer isopachydictyol A were determined to have high contents in a couple of species from the order Dictyotales [18,29]. One of their significant bioactivity potentials is an antithrombotic effect through the inhibition of thrombin [30]. Cembra-4,7,11,15-tetraen-3-ol is a cembranoid-type diterpene that showed good antimicrobial, antitumor, and neuroprotective activities [31].

### 2.3. Headspace Composition of E. amentacea

**Figure 5:**
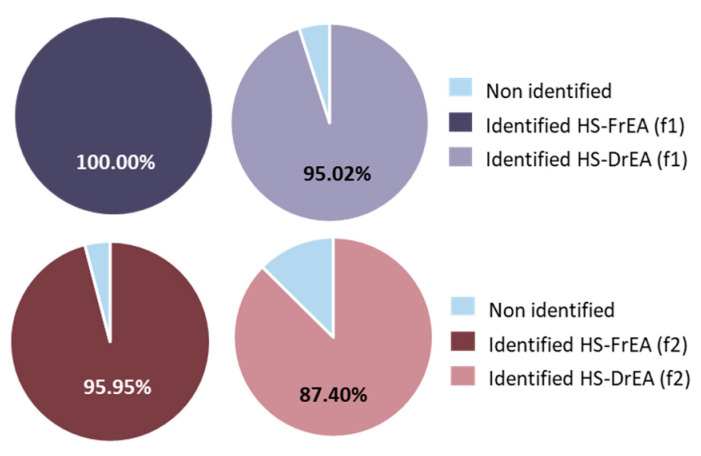
Diagram showing the portion of identified compounds in the headspace of *E. amentacea*. HS-FrEA—headspace of fresh *E. amentacea*; HS-DrEA—headspace of air-dried *E. amentacea*; f1—extracted by DVB/CAR/PDMS fibre; f2—extracted by PDMS/DVB fibre.

The isolation of the headspace VOCs of *E. amentacea* with DVB/CAR/PDMS fibre (f1) provided 100.00% identified compounds in the fresh sample (HD-FrEA) and 95.02% in the dried sample (HD-DrEA). The percentages of the identified compounds when extracted with PDMS/DVB fibre (f2) were 95.95% (HD-FrEA) and 87.40% (HD-DrEA) (Figure 5).

**Figure 6:**
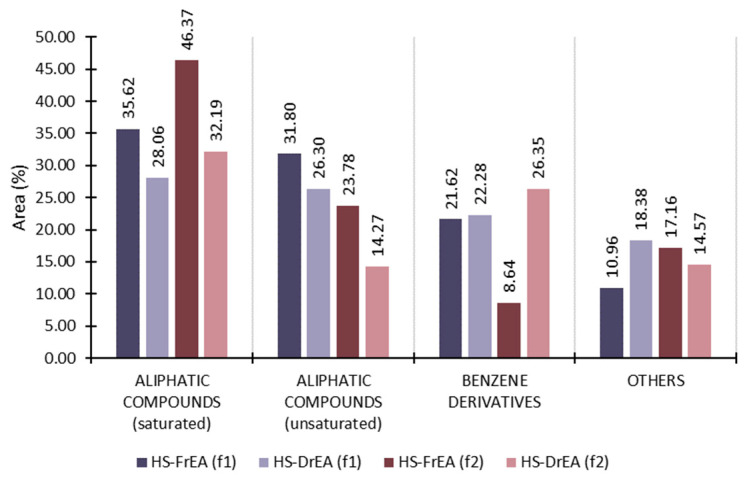
The VOCs of *E. amentacea* extracted by HS-SPME, analysed by GC-MS, and sorted by structural groups. HS-FrEA—headspace of fresh *E. amentacea*; HS-DrEA—headspace of air-dried *E. amentacea*; f1—DVB/CAR/PDMS fibre; f2—PDMS/DVB fibre.

In both the fresh and dry samples isolated with both the f1 and f2 fibres, saturated aliphatic compounds were predominant, showing decrements after the process of drying (Figure 6). Unsaturated aliphatic compounds, as the second most represented group (except HS-DrEA, f2), also showed a decrement after drying (Figure 6). This trend was mostly due to the evaporation of C5 and C6 alcohols and aldehydes, which were highly abundant in HS-FrEA and significantly or completely decreased in HS-DrEA (Table 3). The biggest differences were for hexan-1-ol (HS-FrEA; 12.64%, f1; 12.36%, f2) and (*Z*)-hex-3-en-1-ol (HS-FrEA; 10.15%, f1; 10.75%, f2) since they were not detected in HS-DrEA (or detected in traces with f2). Pentadecane and heptadecane were the most abundant hydrocarbons, and they increased after the drying, which was the result of the degradation of fatty acids.

The greatest difference between fibres was noticed in the group of benzene derivatives (Figure 6). When extracting with f1 there was a minor increment in benzene derivatives in HS-DrEA compared to HS-FrEA. The extraction with f2 showed their lower percentage in HS-FrEA than with f1, and after drying there was a great increment. This difference between the fibres was due to the different polarities of the fibres, which created the possibility of different affinities to certain compounds. The area percentage of benzaldehyde was highly different between the fibres (Table 3).

In the group of other compounds, there were carboxylic acids, terpenes, dictyopterenes, compounds containing sulphur, lactone, and furans.

### 2.4. Volatile Oil Composition of E. amentacea

**Figure 7:**
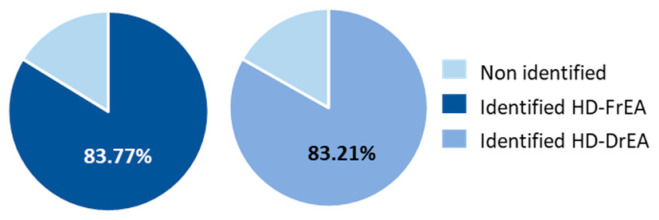
Diagram showing the portion of identified compounds in the hydrodistillate of *E. amentacea*. HD-FrCC—hydrodistillate of fresh *E. amentacea*; HD-DrCC—hydrodistillate of air-dried *E. amentacea*.

When analysing the hydrodistillate of *E. amentacea,* 83.77% of the total compounds were identified in the fresh sample (HD-FrEA), and 83.21% were identified in the air-dried sample (HD-DrEA) (Figure 7).

**Figure 8:**
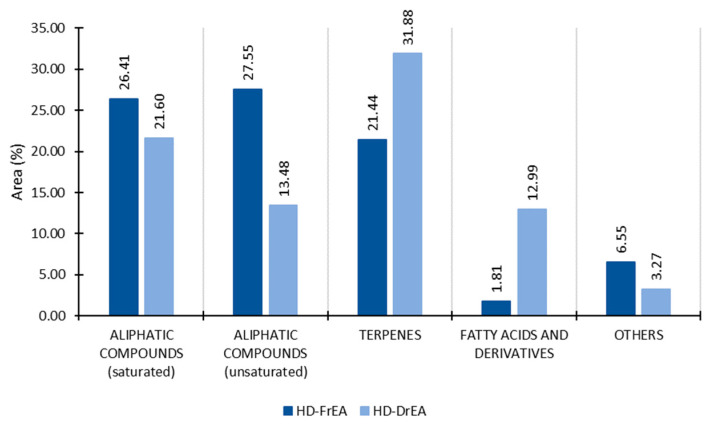
The volatile organic compounds (VOCs) of *E. amentacea* obtained by HD, analysed by GC-MS, and sorted by structural groups. HD-FrCC—hydrodistillate of fresh *E. amentacea*; HD-DrCC—hydrodistillate of air-dried *E. amentacea*.

The content of unsaturated aliphatic compounds (27.55%) in HD-FrEA was slightly greater than the content of saturated aliphatic compounds (26.41%). They both decreased after air drying (2 times, unsaturated; 1.2 times, saturated) (Figure 8). (*E*)-Hex-2-enal was the most abundant among the group of unsaturated aliphatic compounds, with an area percentage of 13.16% in the fresh sample, and was detected in traces in the dry samples. Among the group of saturated aliphatic compounds, pentadecane, heptadecane, pentadecanal, and hexan-1-ol were predominant in terms of abundance in HD-FrEA (Table 4). Their area percentage decreased in HD-DrEA.

The percentage of monoterpenes decreased after drying, but the percentages of diterpenes and especially sesquiterpenes increased, which overall resulted in an increment in terpenes of 1.5 times in HD-FrEA. δ-Selinene (9.66%, HD-FrEA; 16.52%, HD-DrEA) was the major terpene and the most abundant of all compounds in HD-DrEA. Its isomer γ-selinene was detected in traces only in HD-DrEA. Selinene-type sesquiterpenes showed antibacterial and antifungal activities [32].

The greatest increment after drying could be noticed in the group of fatty acids and derivatives. The percentage of hexadecanoic acid increased more than 15 times (Table 4). A great content of hexadecanoic acid was found in the brown alga *Turbinaria ornate*, a member of the order Fucales. It has shown great antioxidant activity as well as an inhibitory effect on HT-29 human colon cancer cells. Thus, it may be a potential anticancer material [33].

In the group of other compounds, there were various compounds such as benzene derivatives, carboxylic acids, phthalates, etc.

## 3. Materials and Methods

### 3.1. Alga Sample

*Cystoseira corniculata* (Turner) Zanardini 1841 and *Ericaria amentacea* (C.Agardh) Molinari and Guiry were collected from the Adriatic Sea (single-point collection). *C. corniculata* was collected in April 2021 in Luka bay on Dugi Otok with the sampling point at the graphical coordinates 43°58′54″ N; 15°05′37″ E. The sea depth was 8 m, while the temperature of the sea was 24 °C. *E. amentacea* was collected at the offshore side of Dugi Otok with the sampling point at the geographical coordinates 43°03′16″ N; 14°59′14″ E. The collection occurred in April 2021 at 0.5 m of the sea depth with the sea temperature at 16 °C. Immediately after the collection, an air-tight plastic box containing both seawater and the algae was transferred to the laboratory. The analysis was carried out after no more than 24 h, and until then the samples were kept at 4 °C in the dark. A part of the collected algae was placed in the dark at room temperature for 10 days to be air-dried. Before further analysis, both fresh and air-dried samples were chopped into smaller pieces. The marine biology experts Donat Ptericiolli and Dr. Tatjana Bakran-Petricioli performed the identification of the collected algae according to [34].

### 3.2. Headspace Solid-Phase Microextraction (HS-SPME)

HS-SPME was performed using a PAL Auto Sampler System (PAL RSI 85, CTC Analytics AG, Zwingen, Switzerland). The extraction of the headspace VOCs was separately carried out on two SPME fibres of different polarities. Both fibres, one covered with DVB/CAR/PDMS (divinylbenzene/carboxen/polydimethylsiloxane) and the other covered with PDMS/DVB (poly-dimethylsiloxane/divinylbenzene), were conditioned for 30 min at 250 °C in a He environment before use and for 5 min between injections. They were both purchased from Agilent Technologies (Palo Alto, Santa Clara, CA, USA). The prepared samples (1 g) were placed into 20 mL glass vials sealed with a polytetrafluorethylene (PTFE)/silicon septa stainless-steel cap. The equilibration of the sample was carried out at 60 °C for 15 min, after which it was extracted for 45 min. The thermal desorption of the fibre was executed directly to the GC column for 6 min at 250 °C. HS-SPME was performed in triplicate for each sample.

### 3.3. Hydrodistillation (HD)

Hydrodistillation was performed for 2 h in a modified Clevenger apparatus. A 3 mL solvent trap composed of pentane and diethyl ether (*v*/*v* ratio 1:2) was put above the water layer in the condensed tube of the apparatus. The solvent trap, containing dissolved VOCs, was then concentrated under a slow nitrogen flow until the final volume of approximately 100 µL. A 2 µL sample was injected for GC–MS analyses.

### 3.4. Gas Chromatography–Mass Spectrometry Analysis of VOCs

A gas chromatograph (8890 Agilent Technologies, Palo Alto, Santa Clara, CA, USA) tandem mass spectrometer detector (model 5977E MSD, Agilent Technologies) was used to analyse VOCs isolated from both *C. corniculata* and *E. amentacea*. The separation of VOCs was performed on an HP-5MS capillary column (30 m × 0.25 mm, 0.25 µm film thickness, Agilent Technologies, Palo Alto, Santa Clara, CA, USA). The GC–MS analysis conditions and the identification procedure of the compounds were specified by Radman et al. [18] The samples were run in triplicate. The retention indices of the compounds were calculated relative to the retention times of n-alkanes (C_9_–C_24_). The compounds were then identified by comparisons of their retention indices (RI) with those reported in the literature (NIST, National Institute of Standards and Technology) and their mass spectra with the spectra from the Wiley9 (Wiley, New York, NY, USA) and NIST 17 (D-Gaithersburg) mass spectral libraries.

## 4. Conclusions

*Cystoseira* species exhibit great biological and chemical diversity that was confirmed by the present research on the headspace and volatile oil composition of *C. corniculata* and *E. amentacea*. HS-SPME and HD were successfully applied to identify the full range of present volatiles (headspace, low, moderate, and less volatile compounds) in fresh and air-dried samples.

The dominant group was the aliphatic compounds in both HS-FrCC and HS-DrCC, with decan-5-ol being the most abundant in HS-FrCC. Although, in general, pentadecane dominates in brown algae, in HS-FrCC it was detected in a very small percentage. Monoterpenes and sesquiterpenes were also present in HS-FrCC. The greatest difference between the fresh and air-dried samples was noted for benzene derivatives, whereas in HS-FrCC their content was remarkably lower. Fatty acids and derivatives were predominant in both fresh and dry samples of the hydrodistillates of *C. corniculata*.

In both fresh and dry samples of *E. amentacea,* saturated aliphatic compounds were predominant, showing a decrement after drying. Pentadecane and heptadecane were the most abundant hydrocarbons, and they increased after drying. (*E*)-Hex-2-enal was the most abundant in the fresh sample and was detected in traces in the dry sample. The greatest increment after drying could be noticed for fatty acids and derivatives.

The great diversity of identified VOCs among those two macroalgae supports their different botanical classifications—*Ericaria amentacea* (C.Agardh) Molinari and Guiry 2020 instead of *Cystoseira amentacea* (C.Agardh) Bory de Saint-Vincent, 1832. Further phytochemical research on these two macroalgae can be encouraged.

## Figures and Tables

**Table 1:** The VOCs from *C. corniculata* that were isolated by HS SPME and analysed by GC–MS: (I—fresh *C. corniculata* extracted by DVB/CAR/PDMS fibre, II—air-dried *C. corniculata* extracted by DVB/CAR/PDMS fibre, III—fresh *C. corniculata* extracted by PDMS/DVB fibre, IV—air-dried *C. corniculata* extracted by PDMS/DVB fibre).

No.	Compound	RI	Area (%) ± SD *			
			I	II	III	IV
1	Dimethyl sulphide	<900	0.71 ± 0.07	1.10 ± 0.35	-	3.68 ± 0.35
2	Hexanal	<900	1.08 ± 0.59	1.75 ± 0.41	0.55 ± 0.07	3.95 ± 0.42
3	(*Z*)-Hex-3-en-1-ol	<900	4.05 ± 0.04	-	6.24 ± 0.063	-
4	Hexan-1-ol	<900	8.12 ± 0.52	-	9.78 ± 0.52	-
5	Heptanal	907	-	0.72 ± 0.16	-	1.38 ± 0.22
6	Benzaldehyde	970	1.01 ± 0.04	1.65 ± 0.11	-	-
7	Oct-1-en-3-ol	984	0.31 ± 0.03	1.04 ± 0.04	0.61 ± 0.10	2.42 ± 0.35
8	Phenol	986	-	1.67 ± 0.07	-	2.34 ± 0.12
9	Octan-3-one	992	0.62 ± 0.04	0.88 ± 0.06	0.48 ± 0.07	1.08 ± 0.02
10	Octan-2-one	995	-	-	0.25 ± 0.02	-
11	(*E*,*Z*)-Hepta-2,4-dienal	996	-	0.61 ± 0.13	-	1.04 ± 0.13
12	Benzyl alcohol	1042	0.33 ± 0.05	17.39 ± 0.53	0.52 ± 0.06	21.35 ± 1.56
13	(*E*)-Oct-2-enal	1064	-	0.95 ± 0.01	-	0.91 ± 0.23
14	Octan-1-ol	1076	0.96 ± 0.07	-	1.18 ± 0.21	-
15	6-[(1*Z*)-Butenyl]-cyclohepta-1,4-diene] (Dictyopterene D’)	1158	0.51 ± 0.09	-	0.40 ± 0.11	-
16	[6-Butylcyclohepta-1,4-diene] (Dictyopterene C’)	1175	0.20 ± 0.03	-	0.17 ± 0.06	-
17	Decan-5-one	1177	4.22 ± 0.14	-	3.00 ± 0.72	-
18	Decan-5-ol	1192	33.56 ± 0.03	-	30.42 ± 1.20	-
19	(*Z*,*E*)-Nona-2,4-dienal	1215	1.61 ± 0.14	-	1.05 ± 0.12	-
20	β-Cyclocitral	1220	5.25 ± 0.52	-	3.18 ± 0.42	-
21	β-Citral	1240	5.25 ± 0.17	-	5.36 ± 0.44	-
22	β-Cyclohomocitral	1262	4.64 ± 0.02	-	5.14 ± 0.63	-
23	Dec-4-en-1-ol	1264	2.53 ± 0.05	-	2.55 ± 0.21	-
24	(*E*,*Z*)-Deca-2,4-dienal	1296	0.47 ± 0.00	0.30 ± 0.02	0.45 ± 0.11	0.68 ± 0.06
25	α-Cubebene	1355	0.34 ± 0.08	1.81 ± 0.09	0.32 ± 0.06	1.40 ± 0.10
26	β-Bourbonene	1389	-	3.28 ± 0.53	-	1.27 ± 0.22
27	β-Cubebene	1392	-	5.26 ± 0.76	-	0.95 ± 0.07
28	γ-Curcumene	1478	0.46 ± 0.10	0.95 ± 0.03	0.31 ± 0.03	0.98 ± 0.21
29	α-Amorphene	1481	-	1.60 ± 0.21	-	1.15 ± 0.35
30	Germacrene D	1485	0.53 ± 0.10	4.57 ± 0.85	0.68 ± 0.02	2.73 ± 0.65
31	epi-Bicyclosesquiphellandrene	1495	0.71 ± 0.03	3.38 ± 0.42	0.73 ± 0.10	1.46 ± 0.40
32	Pentadecane	1500	0.39 ± 0.05	27.62 ± 0.32	0.36 ± 0.04	21.90 ± 0.91
33	Tridecanal	1514	0.50 ± 0.05	-	-	-
34	β-Cadinene	1520	0.61 ± 0.02	0.87 ± 0.38	0.47 ± 0.09	-
35	(*E*)-Calamenene	1528	0.99 ± 0.12	3.37 ± 0.58	0.88 ± 0.30	2.28 ± 0.30
36	γ-Selinene	1530	0.73 ± 0.10	2.25 ± 0.55	0.78 ± 0.23	1.62 ± 0.12
37	(*E*)-Cadina-1,4-diene	1537	0.37 ± 0.05	-	0.29 ± 0.05	-
38	Cubenol	1647	10.37 ± 0.40	5.12 ± 0.68	8.57 ± 0.45	7.90 ± 0.37
39	Heptadecane	1700	0.57 ± 0.16	6.22 ± 0.88	0.26 ± 0.02	9.19 ± 0.90

* SD is the standard deviation of the sample tested in triplicate; RI—retention index.

**Table 2:** The VOCs from *C. corniculata* isolated by HD and analysed by GC–MS: (V—hydrodistillate of fresh *C. corniculata*, VI—hydrodistillate of air-dried *C. corniculata*).

No.	Compound	RI	Area (%) ± SD *	
			V	VI
1	Hex-3-en-1-ol	<899	0.72 ± 0.01	-
2	Hexan-1-ol	<900	0.42 ± 0.00	-
3	Heptan-3-one	<900	-	0.01 ± 0.00
4	Heptanal	903	-	0.01 ± 0.00
5	Benzaldehyde	968	0.01 ± 0.00	0.04 ± 0.01
6	(*Z*)-Octa-1,5-dien-3-one	979	0.01 ± 0.00	-
7	Oct-1-en-3-ol	982	-	0.03 ± 0.00
8	Oct-1-en-3-one	983	0.02 ± 0.00	-
9	6-Methylhept-5-en-2-one	990	-	0.03 ± 0.00
10	Octan-3-one	990	0.01 ± 0.00	-
11	2-Pentylfuran	995	-	0.04 ± 0.00
12	Octan-3-ol	998	0.02 ± 0.00	-
13	Octanal	1005	-	0.01 ± 0.00
14	(*E*)-Oct-2-enal	1063	-	0.01 ± 0.00
15	Acetophenone	1073	-	0.04 ± 0.01
16	(*E*)-Oct-2-en-1-ol	1073	0.02 ± 0.00	-
17	Octan-1-ol	1075	0.09 ± 0.02	-
18	Decan-2-one	1192	0.08 ± 0.00	0.02 ± 0.00
19	(*Z*)-Dec-4-enal	1192	0.59 ± 0.05	-
20	β-Bourbonene	1388	0.06 ± 0.00	0.08 ± 0.00
21	β-Cubebene	1393	0.11 ± 0.01	0.11 ± 0.01
22	β-Elemene	1395	0.07 ± 0.00	-
23	Caryophyllene	1422	0.17 ± 0.02	0.18 ± 0.03
24	(*Z*)-Geranylacetone	1458	0.09 ± 0.01	0.37 ± 0.10
25	γ-Curcumene	1478	0.15 ± 0.02	0.08 ± 0.02
26	α-Amorphene	1481	-	0.05 ± 0.01
27	Germacrene D	1485	0.53 ± 0.06	0.41 ± 0.12
28	(*E*)-β-Ionone	1490	-	0.15 ± 0.03
29	epi-Bicyclosesquiphellandrene	1495	0.54 ± 0.05	0.44 ± 0.07
30	Germacrene C	1497	0.21 ± 0.02	0.32 ± 0.11
31	Pentadecane	1500	0.15 ± 0.02	0.12 ± 0.
32	Germacrene A	1511	0.14 ± 0.03	0.03 ± 0.00
33	Tridecanal	1514	-	0.05 ± 0.01
34	β-Cadinene	1520	0.72 ± 0.11	0.81 ± 0.17
35	(*E*)-Calamenene	1528	0.26 ± 0.05	0.45 ± 0.15
36	γ-Selinene	1530	0.21 ± 0.03	0.24 ± 0.10
37	(*E*)-Cadina-1,4-diene	1537	0.06 ± 0.02	0.04 ± 0.01
38	Dactylol	1562	0.07 ± 0.01	0.05 ± 0.01
39	Nerolidol	1569	-	0.07 ± 0.02
40	Germacrene-4-ol	1578	0.62 ± 0.12	0.18 ± 0.03
41	Gleenol	1589	-	0.13 ± 0.01
42	α-Guaiol	1591	0.45 ± 0.11	0.25 ± 0.06
43	Diethyl phthalate	1595	0.27 ± 0.04	0.28 ± 0.06
44	τ-Cadinol	1633	0.08 ± 0.02	0.11 ± 0.03
45	Cubenol	1649	4.68 ± 0.57	7.77 ± 1.02
46	α-Cadinol	1660	0.43 ± 0.04	1.23 ± 0.10
47	Tetradecan-1-ol	1681	0.30 ± 0.10	0.14 ± 0.04
48	Cadina-1(10),4-dien-8α-ol	1690	0.10 ± 0.02	0.11 ± 0.01
49	Heptadecane	1700	0.16 ± 0.04	0.08 ± 0.01
50	Pentadecanal	1718	0.05 ± 0.00	0.08 ± 0.00
51	Octadec-1-ene	1784	0.04 ± 0.00	-
52	(*Z*,*E*)-Farnesyl acetate	1799	0.09 ± 0.02	0.07 ± 0.02
53	Phytan	1817	0.07 ± 0.01	-
54	Hexadecanal	1833	0.06 ± 0.01	-
55	Hexahydrofarnesyl acetone (phytone)	1850	0.13 ± 0.03	0.15 ± 0.03
56	(*Z*)-Hexadec-11-en-1-ol	1865	0.05 ± 0.01	-
57	Diisobutyl phthalate	1873	0.07 ± 0.02	0.06 ± 0.00
58	Hexadecan-1-ol	1885	-	0.14 ± 0.03
59	(9*Z*)-Hexadeca-1,9-diene	1886	0.10 ± 0.02	-
60	Hexadecanoic acid methyl ester	1919	0.11 ± 0.02	-
61	(5*E*,9*E*)-Farnesyl acetone	1923	0.20 ± 0.01	0.23 ± 0.10
62	Cembrene	1929	0.49 ± 0.07	0.21 ± 0.10
63	Hexadecanoic acid	1979	0.99 ± 0.21	-
64	Eicosane	2000	2.47 ± 0.37	-
65	Methyl octadecyl ether	2032	0.32 ± 0.11	1.60 ± 0.33
66	Methyl (all *Z*) eicosa-5,8,11,14,17-pentaenoate	2041	0.15 ± 0.04	0.15 ± 0.03
67	Methyl (all *Z*) eicosa-5,8,11,14-tetraenoate	2046	0.09 ± 0.02	0.59 ± 0.10
68	(*Z*,*Z*)-Octadeca-9,12-dien-1-ol	2049	1.59 ± 0.11	1.47 ± 0.35
69	(*Z*,*Z*,*Z*)-Octadeca-9,12,15-trien-1-ol	2052	0.51 ± 0.10	0.21 ± 0.02
70	(*Z*)-Octadec-9-en-1-ol	2061	0.90 ± 0.17	0.32 ± 0.07
71	(*Z*,*Z*)-Octadeca-3,13-dien-1-ol	2070	0.15 ± 0.02	0.16 ± 0.05
72	Octadecan-1-ol	2084	1.91 ± 0.31	4.49 ± 0.81
73	(*E*)-Octadec-9-enoic acid methyl ester	2092	0.58 ± 0.08	6.35 ± 0.73
74	(*E*)-Phytol	2117	0.84 ± 0.23	1.03 ± 0.31
75	Pachydictyol A	2121	1.54 ± 0.20	4.34 ± 0.45
76	Methyl octadecanoate	2130	1.43 ± 0.22	0.64 ± 0.22
77	Isopachydictyol A	2136	1.01 ± 0.12	1.47 ± 0.07
78	(*Z*)-Octadec-9-enoic acid (Oleic acid)	2168	0.30 ± 0.08	0.17 ± 0.05
79	(*E*)-Geranylgeraniol	2195	6.51 ± 0.54	6.29 ± 0.60
80	Docosane	2200	2.01 ± 0.19	1.81 ± 0.42
81	Cembra-4,7,11,15-tetraen-3-ol	2231	3.28 ± 0.26	3.32 ± 0.38
82	Diisooctyl phtalate	2275	1.88 ± 0.24	1.53 ± 0.12
83	Methyl eicosanoate	2313	23.88 ± 0.91	23.01 ± 0.70
84	Butyl stearate	2388	14.12 ± 0.96	13.07 ± 1.02

* SD is the standard deviation of the sample tested in triplicate; RI—retention index.

**Table 3:** The VOCs from *E. amentacea* that were isolated by HS-SPME and analysed by GC–MS: (VII—fresh *E. amentacea* extracted by DVB/CAR/PDMS fibre, VIII—air-dried *E. amentacea* extracted by DVB/CAR/PDMS fibre, IX—fresh *E. amentacea* extracted by PDMS/DVB fibre, X—air-dried *E. amentacea* extracted by PDMS/DVB fibre).

No.	Compound	RI	Area (%) ± SD *			
			VII	VIII	IX	X
1	Dimethyl sulphide	<900	2.49 ± 0.58	9.78 ± 0.98	2.06 ± 0.65	0.86 ± 0.12
2	Pent-1-en-3-ol	<900	5.98 ± 0.51	6.37 ± 0.08	3.49 ± 0.75	2.98 ± 0.48
3	Pentanal	<900	6.80 ± 1.07	1.12 ± 0.04	0.59 ± 0.11	-
4	Pentan-3-one	<900	-	-	2.41 ± 0.36	-
5	Pent-2-en-1-ol	<900	8.74 ± 0.38	5.92 ± 0.26	6.16 ± 0.43	2.04 ± 0.16
6	Hexanal	<900	3.16 ± 0.49	3.16 ± 0.17	6.49 ± 0.77	1.75 ± 0.05
7	3-Methylbutanoic acid	<900	-	1.84 ± 0.18	-	1.79 ± 0.10
8	2-Methylbutanoic acid	<900	-	1.32 ± 0.11	-	1.10 ± 0.07
9	(*E*)-Hex-2-enal	<900	-	3.23 ± 0.13	-	1.30 ± 0.11
10	(*Z*)-Hex-3-en-1-ol	<900	10.15 ± 0.77	-	10.75 ± 1.18	-
11	Hexan-1-ol	<900	12.64 ± 0.94	-	12.36 ± 1.26	0.71 ± 0.08
12	Heptan-3-one	<900	-	-	-	3.17 ± 0.69
13	2-Methylpentan-2-ol	<900	-	-	-	1.03 ± 0.20
14	5,5-Dimethylfuran-2-one	961	-	3.00 ± 0.20	-	-
15	Benzaldehyde	970	18.55 ± 1.43	1.86 ± 0.14	3.97 ± 0.76	1.34 ± 0.17
16	Oct-1-en-3-ol	984	4.23 ± 0.20	5.18 ± 0.88	1.84 ± 0.55	2.19 ± 0.33
17	Phenol	985	-	3.57 ± 0.82	1.12 ± 0.06	3.70 ± 0.40
18	6-Methylhept-5-en-2-one	991	-	2.51 ± 0.01	0.75 ± 0.12	1.78 ± 0.18
19	2-Pentylfuran	996	1.40 ± 0.17	1.43 ± 0.04	1.08 ± 0.06	1.10 ± 0.05
20	2-Ethylhexan-1-ol	1034	1.46 ± 0.15	-	1.49 ± 0.26	2.54 ± 0.29
21	Benzyl alcohol	1041	2.35 ± 0.18	13.60 ± 1.06	2.63 ± 0.07	15.06 ± 1.54
22	Phenylacetaldehyde	1051	0.72 ± 0.11	1.43 ± 0.18	0.93 ± 0.13	1.49 ± 0.19
23	γ-Caprolactone (5-ethyloxolan-2-one)	1061	-	1.00 ± 0.32	-	-
24	Octylcyclopropane	1076	-	-	1.63 ± 0.25	1.53 ± 0.14
25	(*E*,*E*)-Octa-3,5,dien-2-one	1098	2.70 ± 0.04	-	-	-
26	Nonanal	1108	-	-	0.91 ± 0.26	-
27	2-Phenylethanol	1120	-	1.83 ± 0.29	-	1.38 ± 0.25
28	6-[(1*Z*)-Butenyl]-cyclohepta-1,4-diene] (Dictyopterene D’)	1158	3.14 ± 0.26	-	5.19 ± 0.71	0.95 ± 0.09
29	[6-Butylcyclohepta-1,4-diene] (Dictyopterene C’)	1175	0.74 ± 0.04	-	1.08 ± 0.26	-
30	β-Cyclocitral	1226	0.92 ± 0.09	-	0.55 ± 0.16	-
31	Undecan-2-one	1297	-	-	-	0.69 ± 0.07
32	(*E*,*E*)-Deca-2,4-dienal	1320	-	-	0.80 ± 0.10	-
33	2-(2-Butoxyethoxy)ethyl acetate	1372	2.27 ± 0.09	-	2.54 ± 0.24	1.97 ± 0.05
34	Tetradecane	1400	-	1.73 ± 0.11	-	1.26 ± 0.12
35	Pentadecane	1500	6.95 ± 0.42	11.21 ± 1.02	10.73 ± 0.91	10.87 ± 0.80
36	δ-Cadinene	1509	-	-	3.03 ± 0.59	5.28 ± 0.74
37	Tridecanal	1514	1.11 ± 0.25	-	3.33 ± 0.54	0.91 ± 0.27
38	2,4-Di*tert*-butylphenol	1518	-	-	-	3.38 ± 0.82
39	(*E*)-Heptadec-8-ene	1690	-	1.64 ± 0.11	-	1.72 ± 0.09
40	Heptadec-1-ene	1696	-	1.45 ± 0.14	-	1.59 ± 0.06
41	Heptadecane	1700	3.50 ± 0.13	10.84 ± 0.74	5.55 ± 0.90	9.95 ± 1.03
42	Pentadecanal	1718	-	-	1.54 ± 0.02	-
43	Hexadecanal	1833	-	-	0.97 ± 0.16	-

* SD is the standard deviation of the sample tested in triplicate; RI—retention index.

**Table 4:** The VOCs from *E. amentacea* isolated by HD and analysed by GC–MS: (XI—hydrodistillate of fresh *E. amentacea*, XII—hydrodistillate of air-dried *E. amentacea*).

No.	Compound	RI	Area (%) ± SD *	
			XI	XII
1	2-Methylbutanoic acid	<900	-	0.08 ± 0.01
2	(*E*)-Hex-2-enal	<900	13.16 ± 0.96	0.56 ± 0.11
3	Norsabinane	<900	0.34 ± 0.09	-
4	4-Methyloctane	<900	-	0.02 ± 0.00
5	Hexan-1-ol	<900	3.64 ± 0.66	0.03 ± 0.00
6	Heptan-2-one	<900	0.15 ± 0.03	0.04 ± 0.01
7	Nonane	900	1.92 ± 0.42	1.23 ± 0.36
8	Heptanal	903	0.27 ± 0.05	-
9	(*E*,*Z*)-Hexa-2,4-dienal (Sorbaldehyde)	914	0.09 ± 0.01	-
10	(5*E*)-3-Ethylocta-1,5-diene	950	0.35 ± 0.11	-
11	(*E*)-Hept-2-enal	961	0.12 ± 0.03	-
12	Benzaldehyde	967	0.42 ± 0.05	0.09 ± 0.02
13	Pentyl propanoate	978	0.10 ± 0.02	-
14	Oct-1-en-3-ol	982	0.10 ± 0.01	0.03 ± 0.00
15	Octan-2,5-dione	986	0.15 ± 0.03	-
16	6-Methylhept-5-en-2-one	987	0.32 ± 0.08	-
17	2-Pentylfuran	994	0.67 ± 0.12	0.12 ± 0.04
18	2-[(*E*)-Pent-1-enyl]furan	1000	0.67 ± 0.10	0.15 ± 0.03
19	(*E*,*E*)-Hexa-2,4-dienal	1014	0.25 ± 0.06	0.03 ± 0.00
20	2-Ethylhexan-1-ol	1033	0.08 ± 0.01	-
21	1,8-Cineole	1039	0.03 ± 0.00	-
22	Benzyl alcohol	1040	-	0.06 ± 0.01
23	Phenylacetaldehyde	1050	0.25 ± 0.05	0.14 ± 0.04
24	(*E*)-Oct-2-enal	1063	0.21 ± 0.03	0.10 ± 0.02
25	Acetophenone	1072	0.14 ± 0.03	0.06 ± 0.01
26	Octylcyclopropane	1076	0.35 ± 0.12	0.06 ± 0.00
27	1-Methylsulfanylpentan-3-one	1090	1.37 ± 0.40	0.09 ± 0.02
28	Nonan-2-one	1095	0.08 ± 0.02	0.04 ± 0.00
29	(*E*,*Z*)-Octa-3,5-dien-2-one	1097	0.57 ± 0.13	0.05 ± 0.00
30	Linalool	1103	0.12 ± 0.03	0.03 ± 0.00
31	Nonanal	1105	0.09 ± 0.02	0.05 ± 0.01
32	4-Ketoisophorone	1149	0.07 ± 0.01	0.05 ± 0.01
33	6-[(1*Z*)-butenyl]-cyclohepta-1,4-diene] (Dictyopterene D’)	1158	0.62 ± 0.13	0.12 ± 0.02
34	Nona-3,6-dien-1-ol	1163	0.07 ± 0.01	-
35	[6-Butylcyclohepta-1,4-diene] (Dictyopterene C’)	1174	0.16 ± 0.03	-
36	β-Cyclocitral	1226	0.07 ± 0.01	0.05 ± 0.01
37	Benzothiazole	1228	-	0.06 ± 0.00
38	Decan-1-ol	1277	-	0.10 ± 0.01
39	2,6,11-Trimethyldodecane	1283	-	0.04 ± 0.01
40	Indole	1296	0.39 ± 0.14	0.34 ± 0.06
41	Undecanal	1310	-	0.07 ± 0.01
42	2,4,4-Trimethylcyclopentan-1-ol	1311	0.15 ± 0.03	-
43	(*E*,*E*)-Deca-2,4-dienal	1320	0.48 ± 0.14	0.19 ± 0.05
44	(*E*)-Undec-2-en-1-ol	1347	-	0.08 ± 0.03
45	β-Bourbonene	1389	0.08 ± 0.01	0.10 ± 0.01
46	Tetradec-1-ene	1395	0.26 ± 0.03	0.15 ± 0.01
47	Tetradecane	1400	0.16 ± 0.03	-
48	α-Gurjunene	1405	0.33 ± 0.07	0.37 ± 0.06
49	Dodecanal	1412	0.15 ± 0.02	0.20 ± 0.04
50	α-Santalene	1418	-	0.07 ± 0.01
51	(*Z*)-Geranylacetone	1458	0.63 ± 0.10	0.65 ± 0.09
52	(*E*)-β-farnesene	1466	0.84 ± 0.12	1.18 ± 0.19
53	Dodecan-1-ol	1478	0.81 ± 0.20	0.76 ± 0.15
54	β-Ionone	1489	0.41 ± 0.12	1.10 ± 0.50
55	Pentadec-1-ene	1495	0.65 ± 0.11	0.34 ± 0.09
56	Valencene	1498	1.04 ± 0.06	1.43 ± 0.19
57	Pentadecane	1500	5.51 ± 0.80	0.76 ± 0.12
58	δ-Selinene	1509	9.66 ± 0.76	16.52 ± 0.46
59	Tridecanal	1514	1.70 ± 0.20	1.72 ± 0.14
60	β-Cadinene	1520	1.32 ± 0.07	0.64 ± 0.05
61	δ-Cadinene	1527	-	0.15 ± 0.02
62	γ-Selinene	1533	-	0.26 ± 0.09
63	(*E*)-Cadina-1,4-diene	1536	2.97 ± 0.76	3.00 ± 0.61
64	Nerolidol	1569	-	1.49 ± 0.22
65	Germacrene-4-ol	1580	1.36 ± 0.17	1.15 ± 0.11
66	Tetradecanal	1616	0.27 ± 0.09	0.50 ± 0.10
67	Tetradecan-1-ol	1681	1.12 ± 0.16	1.52 ± 0.09
68	Heptadec-1-ene	1696	0.99 ± 0.23	1.95 ± 0.26
69	Heptadecane	1700	3.41 ± 0.60	1.35 ± 0.32
70	Pentadecanal	1718	3.54 ± 0.31	3.27 ± 0.40
71	(*E*,*E*)-Farnesal	1747	-	0.70 ± 0.17
72	Pentadecan-1-ol	1782	0.17 ± 0.03	0.29 ± 0.05
73	(*Z*)-Hexadec-9-enal	1796	-	0.17 ± 0.03
74	Hexadecanal	1820	1.34 ± 0.20	1.16 ± 0.23
75	Hexahydrofarnesyl acetone (phytone)	1850	0.38 ± 0.07	0.63 ± 0.06
76	*p*-Cumylphenol	1855	0.15 ± 0.02	0.25 ± 0.04
77	(*Z*)-Hexadec-11-en-1-ol	1861	2.75 ± 0.26	0.56 ± 0.16
78	(9*Z*)-Hexadeca-1,9-diene	1866	3.99 ± 0.16	2.02 ± 0.50
79	Diisobutyl phthalate	1873	0.20 ± 0.08	0.51 ± 0.10
80	Hexadecan-1-ol	1884	0.91 ± 0.30	2.69 ± 0.61
81	Nonadec-1-ene	1897	1.37 ± 0.11	1.51 ± 0.14
82	Hexadecanoic acid methyl ester	1923	0.64 ± 0.13	1.18 ± 0.26
83	Hexadecanoic acid	1970	0.53 ± 0.06	7.92 ± 0.72
84	(*Z*)-Octadec-9-enal	1998	0.37 ± 0.09	1.30 ± 0.08
85	Eicosane	2000	0.18 ± 0.05	1.04 ± 0.11
86	Octadecanal	2024	0.74 ± 0.07	2.25 ± 0.36
87	Methyl octadecyl ether	2032	-	1.35 ± 0.45
88	Methyl (all *Z*) eicosa-5,8,11,14,17-pentaenoate	2044	-	1.32 ± 0.14
89	Methyl (all *Z*) eicosa-5,8,11,14-tetraenoate	2049	0.44 ± 0.08	0.76 ± 0.08
90	(*Z*)-Octadec-9-en-1-ol	2055	1.15 ± 0.16	-
91	(*Z*,*Z*,*Z*)-Octadeca-9,12,15-trien-1-ol	2056	0.29 ± 0.03	1.17 ± 0.33
92	(*Z*)-Octadec-9-en-1-ol	2061	-	1.96 ± 0.30
93	14-Methylhexadec-8-yn-1-ol	2078	-	1.30 ± 0.09
94	Octadecan-1-ol	2086	-	1.70 ± 0.40
95	Heptadecanoic acid	2097	-	0.45 ± 0.05
96	Nonadecanal	2104	-	0.77 ± 0.05
97	(*E*)-Phytol	2115	2.59 ± 0.15	2.38 ± 0.70
98	(*Z*)-Octadec-9-enoic acid (Oleic acid)	2168	0.21 ± 0.06	-
99	Geranyl linallol	2193	-	0.23 ± 0.05
100	Cembra-4,7,11,15-tetraen-3-ol	2234	-	0.86 ± 0.12

* SD is the standard deviation of the sample tested in triplicate; RI—retention index.

## Data Availability

Not applicable.

## Sample Availability

Samples of the compounds are not available from the authors.

## Abbreviations

VOCs	volatile organic compounds
GC-MS	gas chromatography–mass spectrometry
HS-SPME	headspace solid-phase microextraction
HD	hydrodistillation
DVB/CAR/PDMS	divinylbenzene/carboxene/polydimethylsiloxane
PDMS/DVB	polydimethylsiloxane/divinylbenzene
f1	DVB/CAR/PDMS fibre
f2	PDMS/DVB fibre
FrCC	fresh *Cystoseria corniculata*
DrCC	dry *Cystoseria corniculata*
FrEA	fresh *Ericaria amentacea*
DrEA	dry *Ericaria amentacea*
HS-FrCC	headspace composition of fresh *C. corniculata*
HS-DrCC	headspace composition of dry *C. corniculata*
HS-FrEA	headspace composition of fresh *E. amentacea*
HS-DrEA	headspace composition of dry *E. amentacea*
HD-FrCC	hydrodistillate of fresh *C. corniculata*
HD-DrCC	hydrodistillate of dry *C. corniculata*
HD-FrEA	hydrodistillate of fresh *E. amentacea*
HD-DrEA	hydrodistillate of dry *E. amentacea*
PUFA	polyunsaturated fatty acids
MUFA	monounsaturated fatty acids
FAME	fatty acid methyl esters
